# Evaluation of four environmental sampling methods for the recovery of multidrug-resistant organisms

**DOI:** 10.1017/ash.2023.232

**Published:** 2023-09-29

**Authors:** Ahmed Babiker, Alex Page, Julia Van Riel, Eli Wilber, Amanda Strudwick, Chris Bower, Michael Woodworth, Sarah Satola

## Abstract

**Background:** Environmental contamination is a major risk factor for multidrug-resistant organism (MDRO) exposure and transmission in the healthcare setting. Sponge-stick sampling methods have been developed and validated for MDRO epidemiological investigations, leading to their recommendation by public health agencies. However, similar bacteriological yields with more readily available methods that require less processing time or specialized equipment have also been reported. We compared the ability of 4 sampling methods to recover a variety of MDRO taxa from a simulated contaminated surface. **Methods:** We assessed the ability of (1) cotton swabs moistened with phosphate buffer solution (PBS), (2) e-swabs moistened with e-swab solution, (3) cellulose-containing sponge sticks (CSS), and (4) non–cellulose-containing sponge sticks (NCS) to recover extended-spectrum β-lactamase (ESBL)–producing *Escherichia coli*, carbapenem-resistant *Pseudomonas aeruginosa* (CRPA), carbapenem-resistant *Acinetobacter baumannii* (CRAB), methicillin-resistant *Staphylococcus aureus* (MRSA), vancomycin-resistant *Enterococcus faecium* (VRE), and a mixture that contained VRE, MRSA, and ESBL organisms. A solution of known bacterial inoculum (~10^5^ CFU/mL) was made for each MDRO. Then, 1 mL solution was pipetted on a stainless-steel surface (8 × 12 inch) in 5 µL dots and allowed to dry for 1 hour. All samples were collected by 1 individual to minimize variation in technique. Sponge sticks were expressed in PBS containing 0.02% Tween 80 using a stomacher, were centrifuged, and were then resuspended in PBS. Cotton and e-swabs were spun in a vortexer. Then, 1 mL of fluid from each method was plated to selective and nonselective media in duplicate and incubated at 35°C for 24 hours (MRSA plates, 48 hours) (Fig. 1). CFU per square inch and percentage recovery were calculated. **Results:** Table 1 shows the CFU per square inch and percentage recovery for each sampling method–MDRO taxa combination. The percentage recovery varied across MDRO taxa. Across all methods, the lowest rate of recovery was for CRPA and the highest was for VRE. Regardless of MDRO taxa, the percentage recovery was highest for the sponge stick (CSS and NCS) compared to swab (cotton and E-swab) methods across all taxa (Table 1 and Fig. 2).

**Conclusions:** These findings support the preferential use of sponge sticks for the recovery of MDROs from the healthcare environment, despite the additional processing and equipment time needed for sponge sticks. Further studies are needed to assess the robustness of these findings in noncontrived specimens as well as the comparative effectiveness of different sampling methods for non–culture-based MDRO detection.

**Figure:**
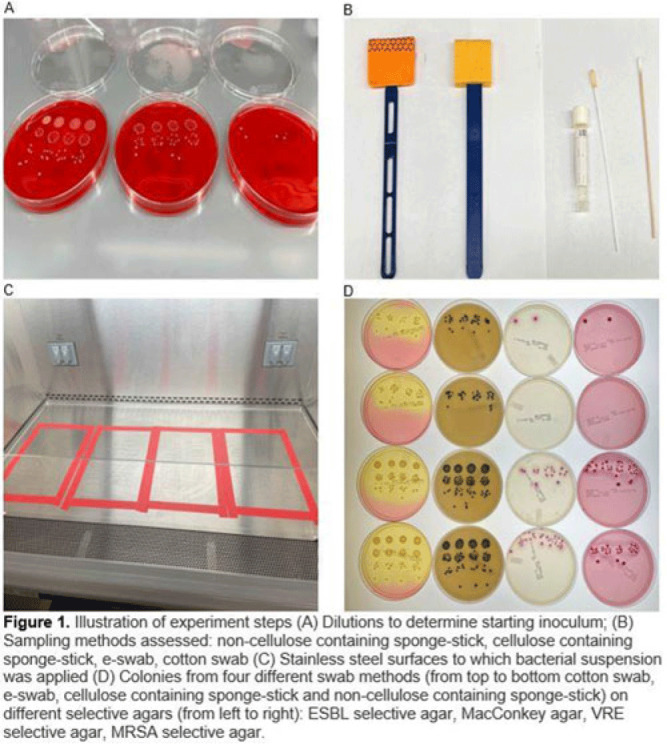

**Figure:**
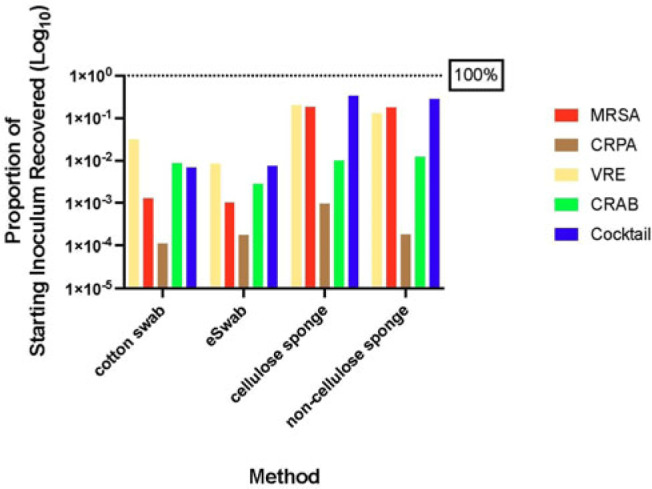

**Figure:**
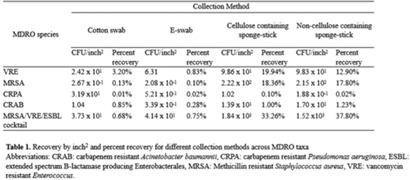

**Disclosure:** None

